# Supplementary figures and images for: First use of molecular evidence to match sexes in the Monstrilloida (Crustacea: Copepoda), and taxonomic implications of the newly recognized and described, partly Maemonstrilla-like females of Monstrillopsis longilobata Lee, Kim & Chang, 2016
Source: PeerJ. 2018 Jun 13;6:e4938. doi: 10.7717/peerj.4938 (PMC6004111; doi:10.7717/peerj.4938)

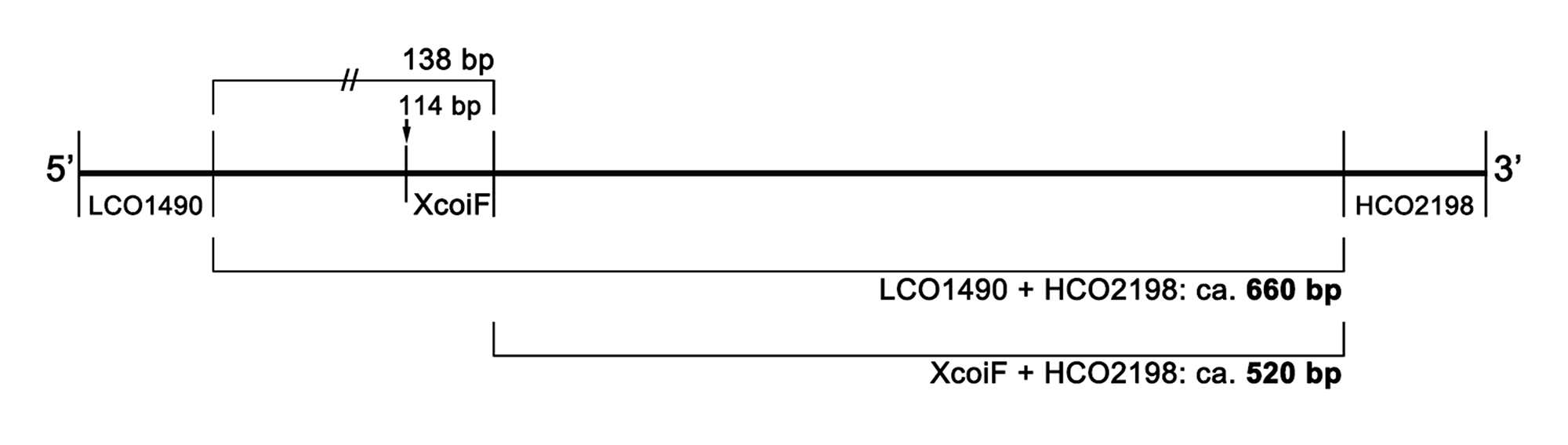

Supplement: Supplemental Information 1 — A total of 24 mtCOI genes covering five genera, and eight species of monstrilloids sharing a conserved region at the base position (bp) of 114th to 138th within the sequences determined using LCO1490 and HCO2198. [file peerj-06-4938-s001.png]

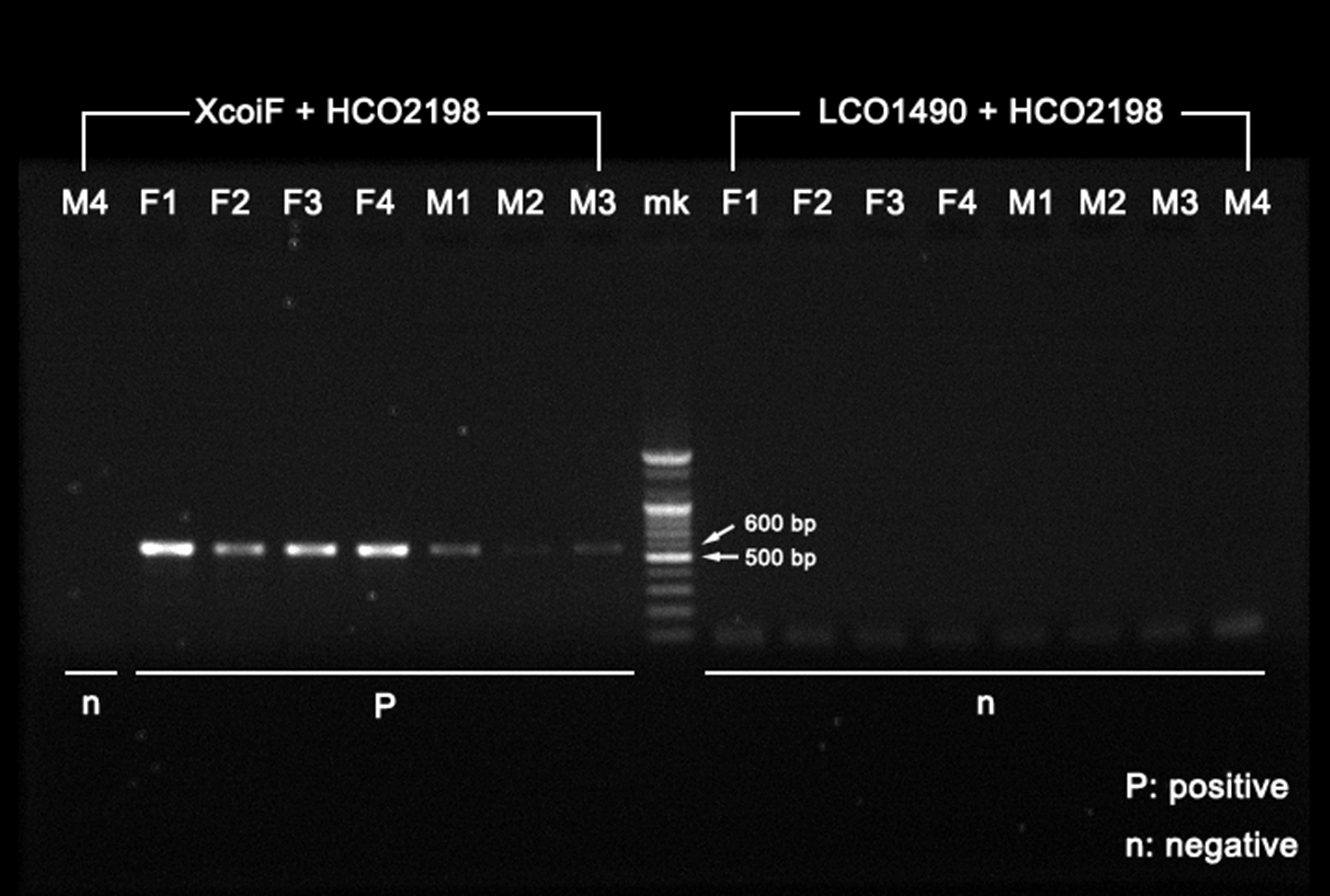

Supplement: Supplemental Information 2 — “XcoiF + HCO2198” primer set results in about 520 bp products as expected (on the left), whereas “LCO1490 + HCO2198” primer set failed proper amplifications (on the right). Four of each female and male specimen used. Specimen numbers given in combination of sex indication (F: female; M: male) and individual distinguishing number; mk: marker (100 bp Ladder, Bioneer). [file peerj-06-4938-s002.png]
