# Supplementary material for: First use of molecular evidence to match sexes in the Monstrilloida (Crustacea: Copepoda), and taxonomic implications of the newly recognized and described, partly Maemonstrilla-like females of Monstrillopsis longilobata Lee, Kim & Chang, 2016
Source: PeerJ. 2018 Jun 13;6:e4938. doi: 10.7717/peerj.4938 (PMC6004111; doi:10.7717/peerj.4938)
Supplement: Supplemental Information 3 [file peerj-06-4938-s003.docx]

| **Antennular segment** | **Proposed nomenclatural marks for setal elements** | **Element type** | ***Monstrilla grandis* ♀** | ***Monstrilla ilhoii* ♀** | ***Monstrilla ilhoii* ♂** | ***Cymbasoma striifrons* ♀** | ***Monstrillopsis longilobata* ♀** | ***Monstrillopsis longilobata* ♂** | ***Monstrillopsis coreensis* ♂** | ***Caromiobenella castorea* ♂** | ***Caromiobenella polluxea* ♂** | ***Caromiobenella hamatapex* ♀** |
| --- | --- | --- | --- | --- | --- | --- | --- | --- | --- | --- | --- | --- |
| **1st segment** | 1 | Spine or setiform seta**^N1^** | O | O | O | O | O | O | O | O | O | short, naked seta |
| **2nd segment (or purported 2nd)** | 2d_1_ | Spine | 2d_1-3_, 2v_1-2_* | O | O | O | O | O | O | O | O | five spines,  single seta |
|  | 2d_2_ | Spine or setiform seta **^N2^** |  | O | O | O | O | O | O | O | O |  |
|  | 2v_1_ | Spine |  | O | O | O | O | O | O | O | O |  |
|  | 2v_2_ | Spine |  | O | O | O | O | O | O | O | O |  |
|  | 2v_3_ | Spine or setiform seta **^N3^** |  | O | O | O | O | O | O | O | O |  |
|  | IId | Seta | O | O | O | O | O | O | O | O | O |  |
| **3rd segment (or purported 3rd)** | 3 | Spine | O | O | O | O | O | O | O | O | O | two setae,  single spine |
|  | IIId | Seta | O | O | O | O | O | O | O | O | O |  |
|  | IIIv | Seta | O | O | O | O | O | O | O | O | O |  |
| **4th segment (or purported 4th)** | 4d_1_ | Spine | O | O | O | O | O | O | O | O | O | five spines,  two setae;  "4a" not present |
|  | 4d_2_ | Spine | O | O | O | O | O | O | O | O | O |  |
|  | 4a | Spine | X | 4d_3_ | arrowed | X | X | *** | *** | 4d_a_ | 4d_a_ |  |
|  | 4v_1_ | Spine | O | O | O | O | O | O | O | O | O |  |
|  | 4v_2_ | Spine | O | O | O | O | O | O | O | O | O |  |
|  | 4v_3_ | Spine | O | O | O | O | O | O | O | O | O |  |
|  | IVd | Seta | O | O | O | O | O | X | X | X | X |  |
|  | IVv | Seta | O | O | X | O | O | O | O | O | O |  |
|  | 4aes | Aesthetasc | O | O | O | O | O | O | O | O | O | O |
| **5th segment (or purported 5th)** | 5_1_ | Spine | 6_1_, 6_2_* | 6_2_ | 2 | 6_1_, 6_2_ * | O | 1 | 1 | 6_1_ | 6_1_ | 6_1_ |
|  | 5_2_ | Spine |  | 6_1_ | 1 |  | O | 2 | 2 | 6_2_ | 6_2_ | 6_2_ |
|  | 5_3_ | Spine | 5 | 5 | 6 | 5 | O | 6 | 6 | 6_3_ | 6_3_ | 5-seta |
|  | 5a | Spine | X | arrowed | ** | X | X | arrowed | arrowed | 7 | 7 | X |
|  | A | Seta | b_1 – 4_ * | b_1_ | O | b_1 – 6_ * | O | O | O | O | O | Six outer distal  b-setae |
|  | B | Seta |  | b_2_ | C |  | O | O | C | O | O |  |
|  | C | Seta |  | b_5_ | B |  | O | D | B | O | O |  |
|  | D | Seta |  | b_3_ | O |  | O | C | O | O | O |  |
|  | a | Seta | b_5_ | b_4_ | 4 |  | O | 4 | 4 | b | b |  |
|  | b | Seta | X | b_6_ | 3 |  | O | 3 | 3 | a | a |  |
|  | Vd | Seta | O | O | 5 | O | O | X | X | X | X | three setae |
|  | Vm | Seta | O | O | X | O | O | X | X | X | X |  |
|  | Vv | Seta | O | O | X | O | O | 5 | 5 | O | O |  |
|  | 5aes | Aesthetasc | X | 6aes | not labeled | 6aes | O | 6aes | 6aes | 6aes | 6aes | 6aes |
| **References** | | | Chang, 2014 | Lee & Chang, 2016 | | Chang, 2012 | This study | Lee, Kim & Chang, 2016 | | Jeon, Lee & Soh, 2018 | | Chang, 2014 |

**O**, element present; labeled, described or both with the same nomenclatural terms of this study.

**X**, element absent; not described nor illustrated in the previous reports.

**Any other marks in the data table**, element present but described and/or illustrated with different nomenclatural terms; terms are given in this table as in original reports.

**^N#^** Element is sometimes rather in setiform in some monstrilloid species.

**^N1^** Setiform element 1 has been often reported from the species of *Cymbasoma* (see Suárez-Morales & McKinnon, 2016; Jeon, Lee & Soh, 2018).

^N2^ Setiform element 2d_2_ has been reported from the species of *Cymbasoma* and *Caromiobenella*.

^N3^ Setiform element 2v_3_ has been reported at least from two *Monstrilla* species, *M*. *inserta* Scott, 1909 and *M*. *brasiliensis* Dias & Suárez-Morales, 2000 (see Suárez-Morales & Dias, 2000).

* Elements that have been checked the presence but failed localization using the proposed terms. This problem is mainly caused by lack of labels and/or detailed descriptions on the position from original studies.

** Element is absent in original description, but has been checked its presence with *Monstrilla ilhoii* Lee & Chang, 2016 from Ulsan, Korea.

*** Element depicted in the illustrations, but not explicitly described and/or mentioned in text.

References

Chang CY. 2012. First record of monstrilloid copepods in Korea: description of a new species of the genus *Cymbasoma* (Monstrilloida, Monstrillidae). *Animal Systematics Evolution and Diversity* 28(2):126–132 DOI 10.5635/ASED.2012.28.2.126.

Chang CY. 2014. Two new records of monstrilloid copepods (Crustacea) from Korea. *Animal Systematics Evolution and Diversity* 30(3):206–214 DOI 10.5635/ASED.2014.30.3.206.

Jeon D, Lee W, Soh HY. 2018. A new genus and two new species of monstrilloid copepods (Copepoda: Monstrillidae): integrating morphological, molecular phylogenetic, and ecological evidence. *Journal of Crustacean Biology* 38(1):45–65 DOI 10.1093/jcbiol/rux095.

Lee J, Chang CY. 2016. A new species of *Monstrilla* Dana, 1849 (Copepoda: Monstrilloida: Monstrillidae) from Korea, including a key to species from the north-west Pacific. *Zootaxa* 4174(1):396–409 DOI 10.11646/zootaxa.4174.1.24.

Lee J, Kim D, Chang CY. 2016. Two new species of the genus *Monstrillopsis* Sars, 1921 (Copepoda: Monstrilloida: Monstrillidae) from South Korea. *Zootaxa* 4174(1):410–423 DOI 10.11646/zootaxa.4174.1.25.

Suárez-Morales E, Dias C. 2000. Two new species of *Monstrilla* (Copepoda: Monstrilloida) from Brazil. *Journal of the Marine Biological Association of the United Kingdom* 80(6):1031–1039 DOI 10.1017/s002531540000309x.
