# Supplementary material for: First use of molecular evidence to match sexes in the Monstrilloida (Crustacea: Copepoda), and taxonomic implications of the newly recognized and described, partly Maemonstrilla-like females of Monstrillopsis longilobata Lee, Kim & Chang, 2016
Source: PeerJ. 2018 Jun 13;6:e4938. doi: 10.7717/peerj.4938 (PMC6004111; doi:10.7717/peerj.4938)
Supplement: Supplemental Information 4 [file peerj-06-4938-s004.docx]

| Base position | 114 | 115 | 116 | 117 | 118 | 119 | 120 | 121 | 122 | 123 | 124 | 125 | 126 | 127 | 128 | 129 | 130 | 131 | 132 | 133 | 134 | 135 | 136 | 137 | 138 |
| --- | --- | --- | --- | --- | --- | --- | --- | --- | --- | --- | --- | --- | --- | --- | --- | --- | --- | --- | --- | --- | --- | --- | --- | --- | --- |
| XcoiF primer sequence (5’-3’; 25mer) | **A** | **T** | **A** | **A** | **C** | **R** | **C** | **T** | **G** | **T** | **A** | **G** | **T** | **A** | **A** | **C** | **T** | **K** | **C** | **T** | **C** | **A** | **Y** | **G** | **C** |
| *Caromiobenella castorea* KY553209 | - | - | - | - | T | G | T | A | - | - | G | - | - | - | - | - | - | T | - | - | - | - | T | - | - |
| *Caromiobenella castorea* KY553210 | - | - | - | - | T | G | T | A | - | - | G | - | - | - | - | - | - | T | - | - | - | - | T | - | - |
| *Caromiobenella polluxea* KY553211 | - | - | - | - | T | G | T | A | - | - | - | - | - | - | - | - | A | T | - | - | - | - | T | - | - |
| *Caromiobenella polluxea* KY553212 | - | - | - | - | T | G | T | A | - | - | - | - | - | - | - | - | A | T | - | - | - | - | T | - | - |
| *Caromiobenella polluxea* KY553213 | - | - | - | - | T | G | T | A | - | - | - | - | - | - | - | - | A | T | - | - | - | - | T | - | - |
| *Monstrilla ilhoii* KY553214 | - | - | - | - | T | A | - | A | A | - | T | - | - | T | - | - | - | G | - | - | - | - | T | - | - |
| *Monstrilla ilhoii* KY553215 | - | - | - | - | T | A | - | A | A | - | T | - | - | T | - | - | - | G | - | - | - | - | T | - | - |
| *Monstrilla ilhoii* KY553216 | - | - | - | - | T | A | - | A | A | - | T | - | - | T | - | - | - | G | - | - | - | - | T | - | - |
| *Monstrilla ilhoii* KY553217 | - | - | - | - | T | A | - | A | A | - | T | - | - | T | - | - | - | G | - | - | - | - | T | - | - |
| *Monstrilla ilhoii* KY553218 | - | - | - | - | T | A | - | A | A | - | T | - | - | T | - | - | - | G | - | - | - | - | T | - | - |
| *Monstrilla* sp.01 KY553219 | - | - | - | - | T | A | - | - | A | - | T | - | - | T | - | - | A | G | - | - | - | - | T | - | - |
| *Monstrilla* sp.01 KY553220 | - | - | - | - | T | A | - | - | A | - | T | - | - | T | - | - | A | G | - | - | - | - | T | - | - |
| *Monstrilla* sp.01 KY553221 | - | - | - | - | T | A | - | - | A | - | T | - | - | T | - | - | A | G | - | - | - | - | T | - | - |
| *Monstrilla* sp.02 KY553222 | - | - | - | - | T | A | - | - | A | - | T | A | - | T | - | - | - | G | - | - | - | - | T | - | - |
| *Monstrilla* sp.02 KY553223 | - | - | - | - | T | A | - | - | A | - | T | A | - | T | - | - | - | G | - | - | - | - | T | - | - |
| *Monstrilla* sp.02 KY553224 | - | - | - | - | T | A | - | - | A | - | T | A | - | T | - | - | - | G | - | - | - | - | T | - | - |
| *Monstrilla* sp.02 KY553225 | - | - | - | - | T | A | - | - | A | - | T | A | - | T | - | - | - | G | - | - | - | - | T | - | - |
| *Cymbasoma* sp.01 KY553226 | - | - | - | - | T | A | - | - | T | - | - | - | - | T | - | - | - | T | - | - | - | - | C | - | - |
| *Cymbasoma* sp.01 KY553227 | - | - | - | - | T | A | - | - | T | - | - | - | - | T | - | - | - | T | - | - | - | - | C | - | - |
| *Cymbasoma* sp.01 KY553228 | - | - | - | - | T | A | - | - | T | - | - | - | - | T | - | - | - | T | - | - | - | - | C | - | - |
| *Monstrillopsis longilobata* KY553229 | - | - | - | - | - | A | - | - | T | - | - | A | - | T | - | - | - | T | - | - | - | - | C | - | - |
| *Monstrillopsis longilobata* KY553230 | - | - | - | - | - | A | - | - | T | - | - | A | - | T | - | - | - | T | - | - | - | - | C | - | - |
| *Maemonstrilla* sp.01 KY553231 | - | - | - | - | T | A | - | - | T | - | - | - | - | T | - | - | A | T | - | - | - | - | T | - | - |
| *Maemonstrilla* sp.01 KY553232 | - | - | - | - | T | A | - | - | T | - | - | - | - | T | - | - | A | T | - | - | - | - | T | - | - |

Degenerate base codes R in 119th bp, K in 131th bp, and Y in 136th bp represent A or G, G or T, C or T, respectively.
